# Influenza Vaccination Coverage among Registered Nurses in China during 2017–2018: An Internet Panel Survey

**DOI:** 10.3390/vaccines7040134

**Published:** 2019-09-29

**Authors:** Jianxing Yu, Xiang Ren, Chuchu Ye, Keqing Tian, Luzhao Feng, Ying Song, Benjamin J. Cowling, Zhongjie Li

**Affiliations:** 1 Division of Infectious Disease, Key Laboratory of Surveillance and Early-Warning on Infectious Disease, Chinese Center for Disease Control and Prevention, Beijing 102206, China; yujianxing@icdc.cn (J.Y.); renxiang@chinacdc.cn (X.R.); tiankq1989@163.com (K.T.); fenglz@chinacdc.cn (L.F.); 2 State Key Laboratory of Infectious Disease Prevention and Control, National Institute for Communicable Disease Control and Prevention, Chinese Center for Disease Control and Prevention, Beijing 102206, China; 3 Research Base of Key Laboratory of Surveillance and Early-Warning on Infectious Disease, Chinese Center for Disease Control and Prevention, Pudong New Area Center for Disease Control and Prevention, Shanghai 200136, China; ccye@pdcdc.sh.cn; 4 Centers for Disease Control and Prevention, Atlanta, GA 30333, USA; kei2@cn.cdc.gov; 5 WHO Collaborating Centre for Infectious Disease Epidemiology and Control, School of Public Health, Li Ka Shing Faculty of Medicine, The University of Hong Kong, Hong Kong Special Administrative Region, Hong Kong, China; bcowling@hku.hk

**Keywords:** public health, health personnel, nurse, influenza vaccines, vaccination, recommendation, internet survey, China

## Abstract

Influenza vaccination is recommended for nurses in China but is not mandatory or offered free of charge. The main objective of this study was to determine influenza vaccination coverage and the principal factors influencing influenza vaccination among nurses in China. During 22 March–1 April 2018, we conducted an opt-in internet panel survey among registered nurses in China. Respondents were recruited from an internet-based training platform for nurses. Among 22,888 nurses invited to participate, 4706 responded, and 4153 were valid respondents. Overall, 257 (6%) nurses reported receiving the seasonal influenza vaccine during the 2017/2018 season. Vaccination coverage was highest among nurses working in Beijing (10%, *p* < 0.001) and nurses working in primary care (12%, *p* = 0.023). The top three reasons for not being vaccinated were lack of time (28%), not knowing where and when to get vaccinated (14%), and lack of confidence in the vaccine’s effectiveness (12%). Overall, 41% of nurses reported experiencing at least one episode of influenza-like illness (ILI) during the 2017/2018 season; 87% of nurses kept working while sick, and 25% of nurses reported ever recommending influenza vaccination to patients. Compared with nurses who did not receive influenza vaccination in the 2017/2018 season, nurses who received influenza vaccination were more likely to recommend influenza vaccination to patients (67% vs. 22%, *p* < 0.001). Influenza vaccination coverage among nurses was low, and only a small proportion recommended influenza vaccine to patients. Our findings highlight the need for a multipronged strategy to increase influenza vaccination among nurses in China.

## 1. Introduction

Vaccination is the most effective method for the prevention of influenza [1], which is associated with 291,000 to 645,000 deaths each year globally [2]. The effectiveness of influenza vaccine varies from season to season, with a pooled efficacy of 59% (95% CI 51%–67%) in adults aged 18–65 years [3], and is mainly influenced by age, underlying medical conditions, as well as the degree of antigenic match between vaccine strains and circulating influenza viruses [4,5,6,7]. In the United States, it was estimated that ~90,000 hospitalizations associated with influenza were prevented by influenza vaccination in the 2013–2014 season, and an additional 42,000 hospitalizations might have been further averted if influenza vaccination coverage was going to reach 70% [8]. The World Health Organization recommends annual influenza vaccination for high-risk groups including older adults, young children, pregnant women, people with underlying medical conditions, and healthcare personnel [9]. As of 2016, more than 115 countries had national influenza immunization policies in place [10]. However, recommendations alone do not translate into high vaccine coverage, and most countries, even those with recommendations, still had low influenza vaccine coverage in 2015 [11]. To build public trust and increase influenza vaccine uptake, policy makers and other decision makers need to better understand vaccination coverage and the factors influencing the acceptance of influenza vaccine among different risk groups [12].

Healthcare personnel (HCP) is one priority group for influenza vaccination [9,13,14,15], as vaccinating HCP can reduce influenza-related morbidity [16,17,18,19,20] and reduce HCP absenteeism [21]. Moreover, HCP play an important role in communicating the benefits of influenza vaccination to their patients [12,22,23]. Maintaining adequate and sustained vaccine coverage among HCP provides a good example for the public and builds public confidence in influenza vaccination. It has been reported that nurses have the highest risk of influenza infection among different occupational groups of HCP [24] and are the public’s most trusted source of information on immunization [12]. In China, influenza vaccination is recommended for HCP but is not mandatory. A handful of cities provide free or subsidized vaccine to older adults and young children [22,25], but HCP are usually not included in these free influenza vaccine policies. Influenza vaccination coverage in China is extremely low, estimated at 1.5%–2.2% among the general population during 2004 and 2014 [25]. An intermediate coverage of 11%–13% for seasonal influenza vaccination was reported among HCP in the influenza pandemic of 2009, during which mass vaccination campaigns were launched in China. However, after the pandemic, the influenza vaccination coverage among HCP dropped back to 5.6% [26,27]. Moreover, China has experienced a series of high-profile vaccine scandals since 2016 [28,29], which reduced public confidence in vaccines. 

We conducted an opt-in internet panel survey among registered nurses in China during the 2017/2018 influenza season. The objective of the study was to determine influenza vaccination coverage and the principal factors influencing influenza vaccination among nurses in China.

## 2. Materials and Methods

### 2.1. Study Design

From 22 March to 1 April 2018, we conducted an opt-in internet survey in six provinces, including Heilongjiang, Beijing, and Shaanxi in northern China; and Guangdong, Yunnan, and Jiangsu in southern China. Provinces were selected to represent the range in socioeconomic development and geographical climate zones across China. We used an internet-based training platform (Hushijia, Beijing Nurselink Technology Co., Ltd., Beijing, China) that provides online training programs to over 650,000 nurses throughout the country. Our study panel consisted of registered nurses who provided their name, professional specialty, contact phone number, WeChat account, or email address in the platform and who worked in one of the six provinces (~226,000 panelists). Invitations containing URL links to the internet survey [30] (English version see also in Supplementary Materials) were sent to a subset of 10% randomly selected panelists through a text messaging service by a focal Nurselink contact in each of the six provinces, a mobile phone application (Hushijia, Beijing Nurselink Technology Co., Ltd., Beijing, China), or a social media chatting software (WeChat, Tencent Holdings, Ltd., Shenzhen, China), depending upon the information the nurse had completed in the platform. Assuming a vaccination coverage rate of 5.6% [27], with a precision level of 1%, a 95% confidence level, and a design effect of two, we planned to collect 4000 valid surveys for all of the six provinces combined. We continued sending invitations to additional nurses in the internet-based platform until our target sample size was achieved. 

### 2.2. Data Collection

We used an electronic questionnaire tool (Wen Juan Xing, Changsha Haoxing Information Technology Co., Ltd., Hunan, China) to design the online questionaire and collect data from panelists. The questionaire (Supplementary Materials) included four components: (i) sociodemographic characteristics of the nurses, including province, age, sex, number of years working in a healthcare institution, type of healthcare institution (e.g., hospital, emergency service center, long-term care facility, blood bank, public health setting, healthcare education institute, pharmacy, etc.), level of hospital care (Chinese health authorities classified hospital into four levels according to hospital’s size and the level of care provided, e.g., tertiary, secondary, primary care, and unclassified), and department (e.g., outpatient clinic, emergency department, internal medicine, surgery, pediatrics, maternity, intensive care unit, and other); (ii) self-reported influenza vaccination in the 2017/2018 season, and the main reason for receiving or not receiving vaccination from a list of possible reasons; (iii) self-reported episodes of influenza-like illness (ILI), defined as reported fever or body temperature ≥ 38 °C and cough or sore throat, experienced during 1 October 2017 and 1 March 2018, and for those with ILI, for the most recent episode, days absent due to ILI, presenteeism or working while sick with ILI, and healthcare-seeking behavior, and (iv) whether the nurse recommended influenza vaccination to patients.

We used screening questions to confirm the eligiblility of visitors to the website. Respondents who did not live within one of the six study provinces or who did not have a nursing license were excluded. Respondents with high levels of missing data (≥50% items missing), who completed the suvey in less than 30 s, or who gave the same answer to 10 consecutive items were also excluded from the study [31,32]. The study protocol and questionnaire were approved by the ethical review committee at the Chinese Center for Disease Control and Prevention (China CDC, Beijing, China).

### 2.3. Statistical Analysis

We calculated influenza vaccination coverage by dividing the number of respondents who reported receiving seasonal influenza vaccine in the 2017/2018 season by the total number of valid respondents. Responses were weighted to the distribution of the Chinese population of nurses by age, sex, province, and type of healthcare institution to compensate for noncoverage and nonresponse. The weights were computed by using an iterative proportional fitting or raking method with the “anesrake” package in R (R Foundation for Statistical Computing, Vienna, Austria) [33]. Demographic data on the population of registered nurses in China were acquired from the National Health Commission of China in 2017 [34]. Influenza vaccination coverage was presented by province and level of healthcare institution. To compare demographic and epidemiological characteristics, we used Chi-square tests or Fisher exact tests for the categorical variables, and Wilcoxon rank-sum or Kruskal–Wallis tests for the continuous variables, as appropriate. Two-sided *p* values of <0.05 were considered statistically significant.

## 3. Results

### 3.1. Characteristics of Study Population

From 22 March to 1 April 2018, we sent 22,888 invitations to nurses in the six provinces, and 4706 (21%) visited the website and completed the survey. The response rates for the three methods of invitation were 77% (3244/4190) for the text messaging service sent by a focal Nurselink contact, 20% (1150/5800) for WeChat, and 2% (322/12,898) for the mobile phone App (Hushijia, Beijing Nurselink Technology Co., Ltd., Beijing, China). After excluding 553 (12%) surveys that did not meet data completeness or quality criteria, a total of 4153 valid surveys were included in our final analysis. Compared with the population of nurses in China overall, our study included larger proportions of nurses aged 25–34 years (60% vs. 48%), and from Beijing (17% vs. 10%), Heilongjiang (19% vs. 9%), and Yunnan (21% vs. 13%) (*p* < 0.001 for all) (Table 1). 

### 3.2. Influenza Vaccination Coverage among Nurses

Overall, 257 (6%) nurses reported receiving influenza vaccination during the 2017/2018 season. Young nurses aged 18–24 years and nurses who had worked for a shorter time (0–2 years) in healthcare sectors had significantly higher coverage (11% for both) compared with other age groups or years of work experience (*p* < 0.001 for both). Among the three levels of healthcare institutions, coverage was highest among nurses working in primary care (12%, *p* = 0.02), followed by secondary care (6%) and tertiary care (5%). Nurses working in outpatient clinics and emergency departments had the highest coverage (13%, *p* < 0.001), while nurses working in surgery had the lowest (3%). Nurses working in Beijing had the highest vaccination coverage (10%, *p* < 0.001), followed by Yunnan (6%), Guangdong (6%), Jiangsu (6%), Shaanxi (3%), and Heilongjiang (3%) (Table 2). 

### 3.3. Reasons for Receiving or not Receiving the Influenza Vaccine in the 2017/2018 Season

**Figure 1:**
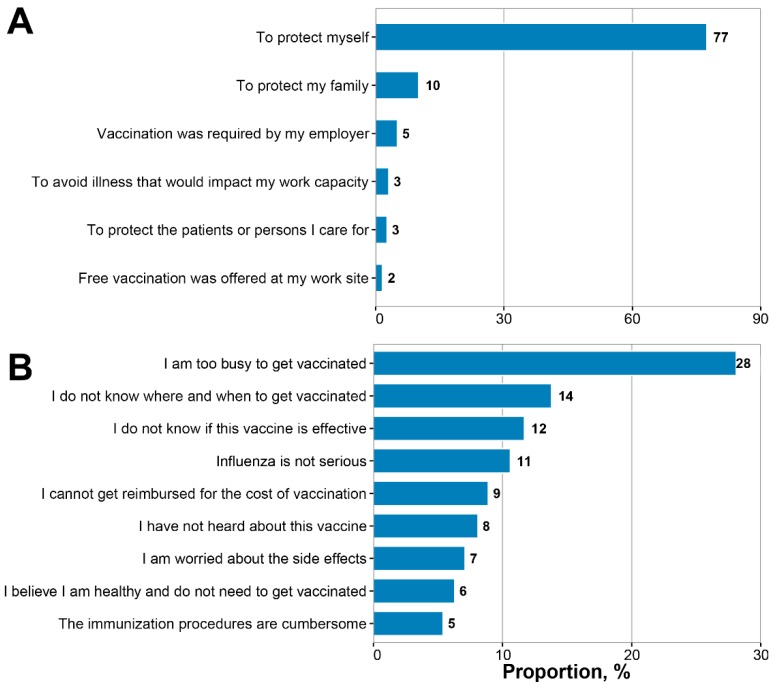
Reasons for receiving or not receiving the influenza vaccine among registered nurses, China, 2017/2018. (**A**) Main reason for receiving the influenza vaccine among 257 vaccinated nurses; (**B**) main reason for not receiving the influenza vaccine among 3896 unvaccinated nurses.

Among the 257 respondents who reported receiving influenza vaccination during the 2017/2018 season, 87% believed that the influenza vaccine offered personal protection or protection for their family. Only 5% of nurses reported that their decision to receive vaccine was because their employer required it, while 2% reported their decision was based on their employer offering free vaccination on site. Other reasons supporting vaccination included protection of patients (3%) and preventing illness that would impact their work capacity (3%) (Figure 1 Panel A). The main reason nurses reported for not receiving influenza vaccine was that they were too busy to get vaccinated (28%). Other important reasons for not receiving vaccination included not knowing where or when to get vaccinated (14%), believing that the influenza vaccine was not effective (12%), believing that influenza did not cause severe illness (11%), and believing that they were not susceptible to influenza infection (6%). Other reasons cited included the financial burden of vaccination (9%), fear of side effects (7%), and cumbersome immunization procedures (5%) (Figure 1 Panel B).

### 3.4. Impact of Vaccination on Self-Reported ILI, Absenteeism, and Presenteeism of Nurses, and on the Likelihood of Nurses Recommending Influenza Vaccination to Patients

During the 2017/2018 season, 41% of nurses (*n* = 1787) reported that they experienced at least one episode of ILI. Among these, 87% (*n* = 1580) continued working while sick, 16% (*n* = 289) reported ever taking sick leave, and 43% (*n* = 752) reported seeking medical care during the course of their latest episode of ILI. Compared with nurses who did not receive influenza vaccination during the 2017/2018 season, nurses who received influenza vaccination were less likely to report ILI (33% vs. 41%, *p*< 0.001) (Table 3). However, sick leave, presenteeism, and healthcare-seeking behavior among nurses were not significantly different between the two comparison groups. 

In total, 25% of nurses (*n* = 1019) recommended influenza vaccination to their patients. Nurses who received influenza vaccination during the 2017/2018 season were more likely to recommend influenza vaccination to their patients (67% vs. 22%, *p* < 0.001) than those who were not vaccinated themselves (Table 3). Nurses aged ≥35 years (32%), working in healthcare sectors ≥6 years (27%), in Beijing (29%), or in primary care (31%) were more likely to recommend influenza vaccine to their patients (*p* < 0.05 for all).

## 4. Discussion

In the 2017/2018 influenza season, the global health community witnessed an upsurge of influenza virus acitivity that posed the greatest impact on the healthcare system since the 2009/10 influenza A(H1N1)pdm09 pandemic [35,36,37,38]. In this study, we report a low coverage of influenza vaccination among registered nurses in China (6%) during the 2017/2018 season, which was considerably lower than coverage among nurses in other countries such as the United States (90.5%) [39], the United Kingdom (68.7%) [40], and other European countries (40%–45%) [41,42]. However, coverage in 2017/2018 was comparable to the national estimates in the 2011/2012 season in China (5.6%) [27], suggesting that limited progress has been made in recent years towards protecting healthcare personnel and their patients from influenza infection through immunization.

Our study found that influenza vaccination coverage among nurses varied by age, number of years working in the healthcare sector, and work setting. Nurses who were aged 18–24 years, had worked for 0–2 years, or who worked in primary care settings had higher vaccination coverage, which is quite similar to findings of a previous study in China [23]. Young nurses who have left school more recently may be more likely to adhere to recommendations on vaccination, and they may also have greater access to health information through new social media technology. Social media can increase the accessibility to knowledge and reduce the system delivery barriers. It has been shown to have a positive effect on the uptake of seasonal influenza vaccine [43]. Moreover, nurses working in primary care settings may have greater access to vaccines, as influenza vaccines in China are usually administered in community health center vaccination clinics, rather than in tertiary- or secondary-level hospitals [23]. Therefore, expanding vaccination clinics from primary healthcare settings to tertiary- and secondary-level hospitals may increase vaccine access to nurses who work in non-primary care settings [39]. 

Moreover, our study observed variations of influenza vaccination coverage among nurses between region. Beijing, a city that introduced a policy that offered free influenza vaccine to older adults and school children since 2007 [22], reported a significantly higher vaccination coverage than the other provinces without free vaccine policies. Free vaccination policies in older adults and children might have led to increased confidence in and acceptance of the influenza vaccine among nurses in Beijing. Even so, the 10% vaccine uptake among Beijing nurses was significantly lower than the coverage of 48.7% among older adults in Beijing [44]. While offering free influenza vaccine has contributed to a consistent rise in vaccine coverage among older adults in Beijing [22], the seasonal influenza vaccine coverage among HCP, a group that does not receive free vaccine, has remained suboptimal. To reduce vaccine coverage disparities between regions and risk groups, strong government commitment, such as policies that offer free or subsidized influenza vaccination, could be assessed and introduced in additional regions and risk groups. 

To build confidence in influenza vaccines, policy and other decision makers benefit from understanding the mixture of scientific, economic, psychological, sociocultural, and political factors influencing the acceptance of influenza vaccine among different risk groups [12]. In our study, the most frequently reported reasons for not being vaccinated were lack of time, not knowing where and when to get vaccinated, lack of confidence in the vaccine’s effectiveness, the belief that influenza does not cause serious illness, and lack of reimbursement for vaccination; while the most common rationale for getting vaccinated among nurses who had received the vaccine was the belief that the influenza vaccine offered protection for themselves or their family. These findings suggest the need for health education/training programs for nurses that communicate HCP’s risk for becoming infected with influenza, the severity of the illness, and the effectiveness and safety of the vaccine. In addition, healthcare employers in China could consider increasing access to vaccination by providing free or subsidized influenza vaccine at the work site, a benefit that only 2% of vaccinated nurses reported receiving in this study. One survey in the United States reported a high coverage of 76% among HCP who worked in locations where free vaccination with no cost to employees and influenza vaccination available on site for >1 day was offered at the worksite by their employer in the 2017–2018 influenza season [39]. Importantly, our study found that only 5% of nurses who received influenza vaccination were required to be vaccinated by their employer. This proportion is low compared with the United States, where influenza vaccination is required for HCP in the majority of healthcare facilities and likely contributes to the high seasonal influenza vaccine coverage rate among nurses (90.5% in 2017/2018 season) [39,45,46]. To protect HCP and possibly their patients from influenza and to reduce healthcare expenditures, requiring influenza vaccination for HCP could be considered [46]. In addition, medical associations in China, including nurses associations [47], could consider formulating a position statement to support the implementation of requirements for influenza vaccination among HCP. 

Our study found that 41% of responding nurses reported an influenza-like illness during the 2017/2018 season. This finding is consistent with one from a previous study that showed that HCP had 3–5 times higher risk of influenza than healthy adults [48]. Of note, among nurses with ILI, 87% reported working while sick, which is slightly higher than previous studies which found that up to three quarters of HCP with ILI continued to work while ill [49]. Presenteeism with ILI among HCP may disrupt patient care, result in higher hospital expenditures [50], and most importantly, pose an increased risk of nosocomial influenza to coworkers and patients, many of whom are at increased risk of severe complications from influenza [51]. Numerous factors might contribute to presenteeism, including heavy workload, not having paid sick leave, or traditional concepts about occupational responsibilities [52]. Hospitals and other healthcare employers could consider implementing paid sick leave and other policies that will encourage HCP with respiratory symptoms to stay at home, to reduce transmission of influenza viruses and other respiratory infections in healthcare settings [50].

HCP’s recommendations for vaccination facilitate vaccine uptake [12,22,23,53]. However, only a quarter of nurses in our study ever recommended influenza vaccination to their patients. This proportion is considerably lower than the four-fifths of healthcare personnel who report recommending influenza vaccine in the United States [54]. Consistent with prior studies [54], nurses who reported being vaccinated in our study were significantly more likely to recommend influenza vaccine to their patients compared with nurses who were not vaccinated (67% vs. 22%). Vaccination of HCP, therefore, not only reduces influenza-related morbidity among HCP and their patients, but also may build vaccine confidence in the public; HCP serve as an example of knowledgeable professionals choosing to get vaccinated, who in turn recommend vaccination to their patients. Indeed, nurses are one of the public’s most trusted sources of information on immunizations [12,23], and nurses’ recommendations for vaccination will increase vaccination coverage among Chinese patients [23]. Training and efforts to encourage HCP in China to provide influenza vaccination recommendations to their patients are needed. 

## 5. Strengths and Limitations

One of the strengths of this study is the survey method, which is easy to implement and replicate, enables access to large and diverse samples, and produces timely results [31,32]. However, this study is also subject to several limitations. First, the opt-in internet panel survey used a nonprobability sample of volunteer nurses from the largest social network of nurses in China, and the response rate among those who were invited was low. Therefore, the study respondents may not adequately represent all nurses in the study provinces. Second, although weights were applied to compensate for noncoverage and nonresponse in our study, only the few risk variables available were adjusted for in our study. Moreover, we only recruited nurses from six provinces. As China is a large country with immense socioeconomic diversity, our results may not be generalisable to nurses residing in other regions or to HCP other than nurses in China. Third, as vaccination status and ILI episodes were self-reported, our findings may be subject to recall bias and repeated entry. Nevertheless, this was the first national effort to utilize available resources, such as the social media network, mobile phone apps, and an internet survey panel, to rapidly monitor and assess influenza vaccine coverage in a population prioritized for influenza vaccination in China. To improve these shortcomings in future study, we will continue monitoring the trends of vaccination coverage in each influenza season, by improving sampling strategy and expanding our panelists to more nurses of other provinces with socialeconomic representativeness of China. We may use incentives to increase the response rate of participants. Moreover, interviewer-based surveys using opt-out probability sampling strategy at selected sites can also be performed to validate the results of the opt-in internet panels survey as it is needed [31].

## 6. Conclusions and Recommendations

Influenza vaccination coverage among nurses in China is low, though the coverage varies by region and population subgroup. Poor availability or accessibility of the vaccine is a major barrier. Our study suggests the need to implement a mutipronged strategy to increase influenza vaccine coverage among nurses in China. This strategy might include the government providing free or subsidized influenza vaccine for HCP, healthcare employers offering on-site vaccination services, instituting occupational requirements for vaccination, and providing health education on the risks of influenza illness and the benefits and safety of influenza vaccination.

## Figures and Tables

**Table 1:** Characteristics of registered nurse internet panel survey respondents, China, 2017/2018.

Characteristics	Chinese Population of Registered Nurses in 2017 *n* = 3,804,021	The Internet Panel of Registered Nurses in Six Provinces, *n* = 226,228	All Respondents, *n* = 4153
Sex			
Male	83,688 (2)	5279 (2)	111 (3)
Female	3,720,333 (98)	220,949 (98)	4042 (97)
Age in years			
<25	513,543 (14)	49,578 (22)	485 (12)
25–34	1,825,930 (48)	162,208 (72)	2505 (60)
≥35	1,464,548 (38)	14,442 (6)	1163 (28)
Type of health care institution			
Hospital	3,591,652 (94)	226,076 (100)	4100 (99)
Tertiary	1,339,414 (37)	186,046 (82)	3329 (80)
Secondary	1,084,090 (30)	37,716 (17)	513 (12)
Primary	176,016 (5)	1711 (1)	166 (4)
Unclassified	992,132 (28)	603 (<1)	92 (2)
Other ^a^	212,369 (6)	152 (<1)	53 (1)
Province			
Beijing	103,459 (10)	73,295 (32)	718 (17)
Heilongjiang	90,446 (9)	37,258 (17)	773 (19)
Shaanxi	126,983 (13)	37,900 (17)	516 (12)
Guangdong	307,664 (31)	22,876 (10)	522 (13)
Jiangsu	236,906 (24)	31,491 (14)	770 (19)
Yunnan	128,513 (13)	23,408 (10)	854 (21)

Note: ^a^ other included emergency service center, long-term care facility, blood bank, public health setting, healthcare education institute, and pharmacy.

**Table 2:** Proportion of registered nurses who received influenza vaccination, China, 2017/2018.

Characteristics	No. in Sample	No. Vaccinated	Weighted % ^#^ Vaccinated	*p*-Value
Age group (years)				<0.001
<25	485	55	11	
25–34	2505	142	6	
≥35	1163	60	5	
Years of work in healthcare sector				<0.001
0–2	455	51	11	
3–5	942	74	7	
≥6	2756	132	5	
Type of health care institution				0.140
Hospital	4100	251	6	
Others ^a^	53	6	11	
Level of healthcare institution				0.023
Tertiary	3329	192	5	
Secondary	513	34	6	
Primary	166	19	12	
Unclassified	145	12	10	
Department				<0.001
Internal medicine	1286	67	5	
Surgery	950	39	3	
Intensive care unit	450	25	5	
Outpatients and emergency department	440	55	13	
Pediatrics and maternity department	198	12	4	
Community health center	171	19	11	
Other	658	40	7	
Province				<0.001
Beijing ^b^	718	77	10	
Heilongjiang	773	31	3	
Shaanxi	516	16	3	
Guangdong	522	30	6	
Jiangsu	770	48	6	
Yunnan	854	55	6	

Note: ^a^ other included emergency service center, long-term care facility, blood bank, public health setting, healthcare education institute, and pharmacy; ^b^ Since 2007, Beijing’s vaccine policy provides free influenza vaccine to older adults and school children; ^#^ numbers in the column were weighted to the distribution of the Chinese population of nurses by age, sex, province, and type of healthcare institution, using an iterative proportional fitting or raking method.

**Table 3:** Association between receipt of influenza vaccination and self-reported influenza-like illness, absenteeism, presenteeism, and healthcare seeking among registered nurses, China, 2017/2018.

Characteristics	Vaccinated Nurse *n* = 257	Unvaccinated Nurse *n* = 3810	Uncertain *n* = 86	*p*-Value
Self-reported influenza-like illness				
Proportion, weighted %	33	41	29	<0.001
Episodes, weighted mean	0.5	0.6	0.5	0.01
Absenteeism				
Proportion, weighted %	17	16	6	0.738
Days, weighted mean	0.2	0.2	0.1	0.233
Presenteeism (e.g., working while sick)				
Proportion, weighted %	81	88	79	0.170
Healthcare seeking				
Proportion, weighted %	46	43	39	0.391
Recommend influenza vaccination to patients				
Proportion, weighted %	67	22	38	<0.001

## Supplementary Materials

The following are available online at https://www.mdpi.com/2076-393X/7/4/134/s1, internet survey: Nurse Flu Survey Questionnaire, China, 2017–2018 Season.
