# Supplementary material for: Influenza Vaccination Coverage among Registered Nurses in China during 2017–2018: An Internet Panel Survey
Source: Vaccines (Basel). 2019 Sep 29;7(4):134. doi: 10.3390/vaccines7040134 (PMC6963313; doi:10.3390/vaccines7040134)
Supplement: Supplementary file 1 [file vaccines-07-00134-s001.pdf]

Nurse Flu Survey Questionnaire, China, 2017–2018 Season

(Translated from original questionnaire in Chinese)

Part I. Basic Information

(1) Sex:
□ Male □ Female

(2) Age:
□ 18–24 years □ 25–29 years
□ 30–34 years □ 35 years and above

(3) Where do you live now?
□ Beijing □ Heilongjiang □ Shaanxi □ Guangdong □ Yunnan □ Jiangsu
□ Other (End survey)

(4) How many years have you been working as a nurse in a healthcare institution?
□ 0–2 years □ 3–5 years □ 6 years and above

(5) Where do you currently work?
□ Hospital/clinic
□ Emergency service center (Jump to Question 8)
□ Long-term care facility (Jump to Question 8)
□ Blood bank (Jump to Question 8)
□ Other, including public health setting, healthcare education institute, and pharmacy etc. (Jump to Question 8)

(6) If you work in a hospital, what is the level of the hospital?
□ Tertiary □ Secondary □ Primary □ Unclassified

(7) If you work in a hospital, what department do you work in?
□ Outpatient clinic □ Fever clinic □ Emergency department □ Surgery
□ Internal medicine (not including the respiratory department)
□ Respiratory department □ Pediatrics
□ Intensive care unit (ICU, CCU, EICU, PICU, NICU, and NSICU etc.)
□ Infectious diseases department □ Immunization clinic
□ Non-clinical departments (including disinfection supply division, medical record room, and laboratory branch etc.)
□ Other

Part II. Self-reported Influenza Like Illness (during the period of 1 October, 2017 to 20 March, 2018)

(8) From 1 October, 2017 to 20 March, 2018, did you have influenza-like illness symptoms (fever with sore throat or cough)?
□ Yes □ No (Jump to Question 12) □ Don't remember (Jump to Question 12)

If yes, I experienced _____episodes of influenza-like illness during this period; among which,

_____episodes of influenza-like illness with body temperature ≥ 38 ° C;

_____episodes of influenza-like illness with body temperature < 38 ° C;

_____episodes of influenza-like illness with body temperature unknown.

(9) During the period of 1 October, 2017 and 20 March, 2018, did you see a doctor for the last episode of your influenza-like illness?

□ Yes □ No (Jump to Question 10) □ Don't remember (Jump to Question 10)

If yes, did your doctor order a test for influenza virus?

□ Yes □ No (Jump to Question 10) □ Don't remember (Jump to Question 10)

If yes, what was the test result?

□ Positive □ Negative

(10) During the period from 1 October, 2017 to 20 March, 2018, did you take sick leave for the last episode of your influenza-like illness?

□ Yes, I took sick leave for___________days. □ No □ Don't remember

(11) During the period from 1 October, 2017 to 20 March, 2018, when you had your last episode of influenza-like illness, did you work while sick?

□ Yes □ No □ Don't remember

(12) Do you think there is a difference between influenza and the common cold?

□ Yes □ No □ Don't know

(13) Do you think the influenza virus can lead to severe consequences (hospitalization, severe complications and death)?

□ Yes □ No □ Don't know

(14) If you have influenza-like illness symptoms (fever with sore throat or cough), will you take the following protections?

Frequent hand washing: □ Yes □ No □ Don't know

Wearing mask: □ Yes □ No □ Don't know

Self-segregation (by avoiding crowded places and close contact with people):

□ Yes □ No □ Don't know

(15) Is the department where you work equipped with a hand sanitizer?

□ Yes □ No

Part III. Self-reported Influenza Vaccination (during the year prior to this survey)

(16) During the year before this survey (i.e. between March, 2017 and March, 2018), were you vaccinated for influenza?

□ Yes □ No (Jump to Question 18) □ Don't remember (Jump to Question 18)

If yes, how was the fee for the influenza vaccine covered?

□ at my own expense □ provided free by my employer □ other

(17) What are the main reasons you were vaccinated for influenza? *(select all that apply)*

□ To protect myself

□ To protect patients or persons I care for

□ To protect my family

□ To avoid the impact illness could have on my capacity to work

□ Vaccination was required by my employer

□ Free vaccination was offered on-site

□ other_______

(18) What are the main reasons you were not vaccinated for influenza? *(select all that apply)*:

□ I don’t know the vaccine

□ I don't know the effect of this vaccine
□ I am worried about the side effects
□ I am too busy to get vaccinated
□ Influenza is not serious
□ I believe I am healthy and do not need to get vaccinated
□ I don't know where and when I can get vaccinated
□ The procedures of vaccination are too cumbersome
□ Vaccination fees cannot be reimbursed
□ other_______

(19)For this year's flu season, are you willing to get vaccinated for influenza?
□ Yes □ No

Part IV. Recommendation of Influenza Vaccine

(20)Have you ever recommended the influenza vaccine to your patients or persons you have cared for?
□ Yes (Jump to Question 22) □ No

(21)If not, what are the main reasons for not recommending the influenza vaccine? *(select all that apply)*
□ I don't know the vaccine
□ I don't know the effect of this vaccine
□ I am worried about the side effects and the subsequent lawsuit raised by patients
□ Influenza is not serious and does not require vaccination
□ other_______

(22)For this year's flu season, for which of the following groups are you willing to recommend influenza vaccine? *(select all that apply)*
□ Children
□ Older adults aged ≥ 65 years
□ Pregnant women
□ Patients with underlying chronic medical conditions
□ Patients who are immunocompromised
□ Adolescents or young adults
□ Family members or friends

Note:

1. Only subjects who obtained a nursing license since January, 2017 will be surveyed.
